# The energy band structure analysis and 2 μm Q-switched laser application of layered rhenium diselenide

**DOI:** 10.1039/c9ra02311a

**Published:** 2019-05-08

**Authors:** Yongping Yao, Xiaowen Li, Rengang Song, Na Cui, Shande Liu, Huiyun Zhang, Dehua Li, Qiangguo Wang, Yan Xu, Jingliang He

**Affiliations:** College of Electrical, Engineering and Automation, Shandong University of Science and Technology Qingdao 266590 China pepsl_liu@163.com x1y5@163.com; College of Electronic and Information Engineering, Shandong University of Science and Technology Qingdao 266590 China; Key Laboratory of Micro-nano Measurement-Manipulation and Physics, Ministry of Education, Department of Applied Physics, Beihang University Beijing 100191 China; State Key Laboratory of Crystal Materials, Shandong University Jinan 250100 China

## Abstract

Layered rhenium diselenide (ReSe_2_) has triggered strong interest because of its outstanding optical and electrical properties. In this paper, we prepared a high-quality multilayer ReSe_2_ saturable absorber with a liquid-phase exfoliation method and characterized its saturable absorption properties around 2 μm. During the Q-switching regime, a maximum average output power of 1.7 W was obtained. A shortest pulse width of 925.8 ns was measured and the corresponding single pulse energy and peak power were 17.6 μJ and 19.0 W, respectively. The results indicate that layered ReSe_2_ is a promising alternative as a nonlinear optical modulator near the 2 μm region.

## Abstract 2

In this paper, we prepared a high-quality multilayer ReSe_2_ saturable absorber with a liquid-phase exfoliation method and characterized its saturable absorption properties around 2 μm.

## Graphical abstract

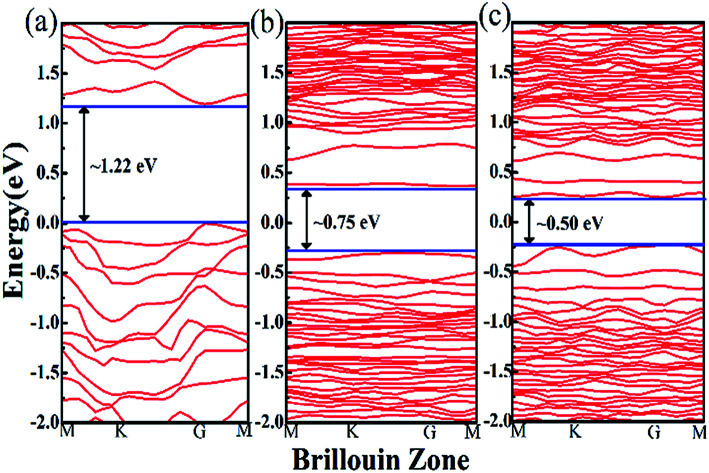

## 1. Introduction

Short laser pulses, especially in the eye-safe range of 2 μm,^1^ have constituted an active research topic in recent years because of their wide variety of applications in the fields of atmospheric gas analysis,^2^ wind lidar,^3^ laser surgery,^4^ and so on. Passive Q-switching (PQS) and mode-locking lasers are the essential technology for generating short laser pulses with pulse width ranging from the nanosecond to femtosecond timescale. In such passive Q-switching lasers, the saturable absorber (SA) is the key element and thus the characteristics of output laser beams strongly depend on the nature of the SA materials. Up to now, semiconductor saturable absorber mirrors (SESAMs),^5^ Cr:ZnS^6^, Cr:ZnSe^7^, carbon nanomaterials,^8,9^ topological insulators (TI)^10,11^ and black phosphorus (BP),^12^ have been recognized as reliable SAs for 2 μm lasers. However, the utilization of SESAMs is restricted by reasons of the complicated and expensive fabrication process as well as narrow absorption bandwidth. The Cr-doped crystals, like Cr:ZnS and Cr:ZnSe, have played pivotal roles at 2 μm region lately.^13^ Nevertheless, the lack of quality and high-cost are the key factor which constrain them further development. The extraordinary performances such as high carrier mobility, easy-preparation, cost-effective, remarkable electrical and thermal conductivity make the graphene attractive candidate as SAs during the PQS regime. But the low absorption efficiency may limit its applications. Hence, more efforts should be made to explore novel nonlinear optical materials for SAs in the 2 μm region.

Owing to possessing excellent saturable absorption properties and environmental stability, transition metal dichalcogenides (TMDs), which have layered structure of the X–M–X form (where M denotes a transition metal and X denotes a chalcogen), have been achieved encouraging results at 2–3 μm laser operations.^14–19^ Rhenium diselenide (ReSe_2_), one of the relatively unexplored members of the TMDs family, has attracted a significant attention because of its unusual electronic and optical behaviors.^20–24^ It crystallizes in a distorted octahedral (1T) diamond-chain structure at the triclinic symmetry. It demonstrates anisotropic response arising from its in-plane anisotropic structure. Rhenium atom contain an extra electron in the d-orbitals,^25,26^ which is different from other 2H phase TMDs (*e.g.*, MoS_2_, WS_2_). ReSe_2_ films can be easily exfoliated from its bulk crystal because of the relatively weaker van der Waals forces between interlayers. The recovery time of ReSe_2_ on the order of 10 ps, which is beneficial for the ultrafast pulse generation. In addition, the layered ReSe_2_ samples show no obvious signs of degradation while exposed to exterior circumstance for several weeks in our study. All the characteristics mentioned above indicate that the layered ReSe_2_ is an excellent material for nonlinear optical modulate and it has been applied as SA at 1–2 μm regions.^27–29^ In 2019, Li *et al.* reported a ReSe_2_ Q-switched Tm:YLF laser, however, the laser pulse width and pulse repetition rate were only 1.61 μs and 28.78 kHz, respectively.^29^ According to excellent properties of the ReSe_2_ material, the laser pulse characteristics at 2 μm would be further improved.

In this paper, a high-quality large-area multilayer ReSe_2_ SA was successfully prepared and the saturable absorption properties at 2 μm region were investigated by the open-aperture Z-scan method. Based on ReSe_2_ as SA, a stable all-solid-state Q-switched Tm:YAP crystal laser was realized. Under an absorbed pump power of 8.5 W, a continuous-wave (CW) output power of 2.9 W was generated, corresponding to a slope efficiency of 44%. In the ReSe_2_ Q-switching regime, a maximum average output power of 1.7 W was obtained, giving a slope efficiency of 37%. The shortest pulse duration of 925.8 ns was generated at a repetition rate of 89.4 kHz, resulting in the maximum single pulse energy and peak power of 17.6 μJ and 19.0 W, respectively.

## 2. Theoretical analysis of the energy band structure

A monolayer supercell of ReSe_2_ with 16 Re atoms and 32 Se atoms and the same structures with one and two Se atomic vacancies are constructed. The ratios (*R*) between Re and Se atoms for the three structures are 1 : 2, 1 : 1.937 and 1 : 1.875. The first principle calculation of band structure is based on the Vienna ab initio simulation package (VASP).^30^ The Perdew–Burke–Ernzerhof (PBE) functional within the generalized gradient approximation (GGA) is adopted to account for the exchange and correlation part.^31^ A plane wave basis set with an energy cutoff of 550 eV and the PAW pseudopotential implemented in VASP are applied. The Brillouin zone is sampled by a Monkhorst–Pack *k*-point mesh of 21 × 21 × 1. In order to avoid the interactions among ReSe_2_ layers, the vacuum space thickness along the *c* axis is set to 20 Å.

**Fig. 1:**
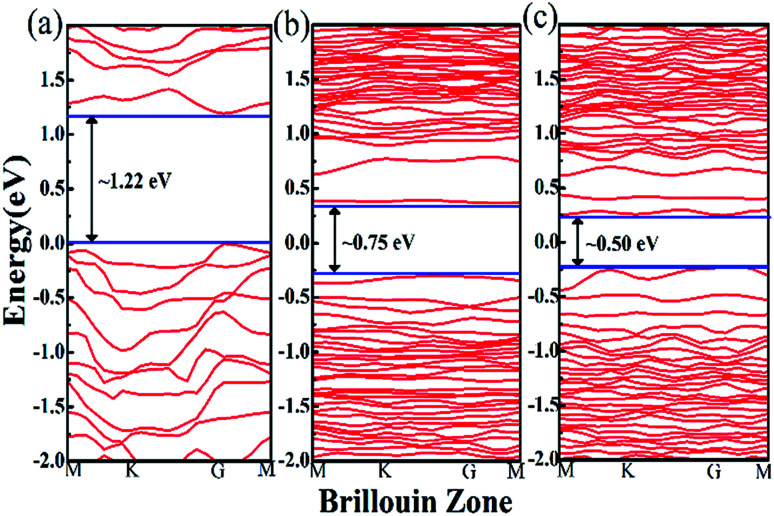
Theoretical band structures for (a) *R* = 1 : 2, (b) *R* = 1 : 1.937, and (c) *R* = 1 : 1.875.

**Fig. 2:**
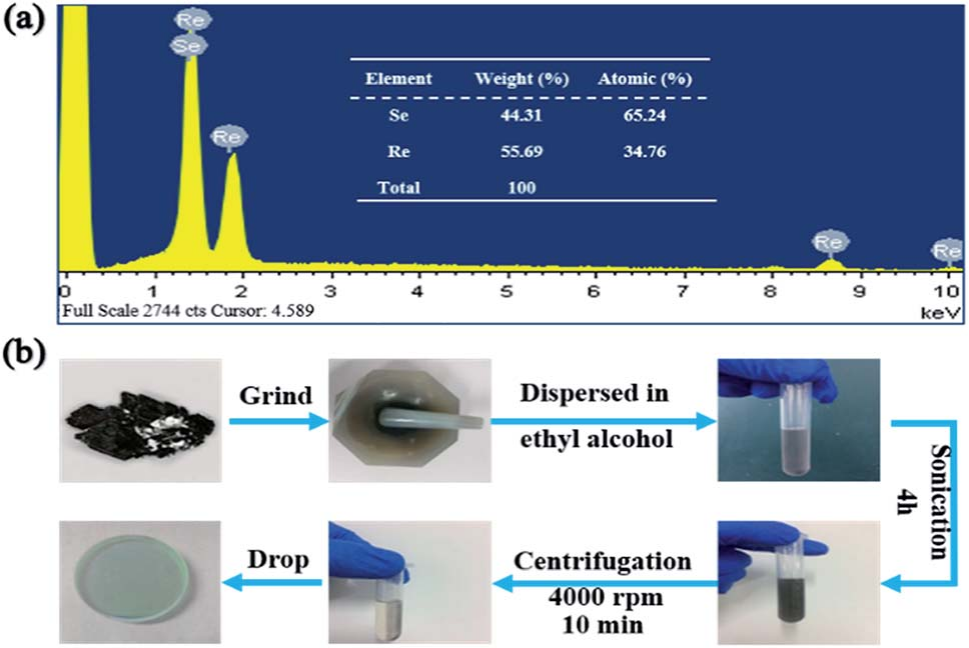
(a) EDS image and the corresponding Re to Se radio of the ReSe_2_ sample, (b) fabrication process of ReSe_2_-SA.

The calculated band structures corresponding to the atomic structures mentioned above are shown in Fig. 1(a–c) respectively. From Fig. 1(a), it can be seen that the monolayer supercell ReSe_2_ with no vacancy has a bandgap of ∼1.22 eV, which is in good agreement with previous calculations of ReSe_2_.^25^ While in Fig. 1(b) and (c), the bandgaps of the structures with one and two Se atomic vacancies are ∼0.75 eV and ∼0.50 eV respectively. With the increase of Se atomic vacancies, the bandgap of monolayer ReSe_2_ decreases from 1.22 eV to 0.50 eV, mainly due to the reconstruction of atoms around the vacancies perturbs the periodic potential. The corresponding absorption wavelength edge for the structures mentioned above extends from 1 μm to 2.48 μm. To identify the existence of the vacancies in the ReSe_2_ sample, the energy-dispersive X-ray spectroscopy (EDS) was measured. Fig. 2(a) presents the ratio of Re to Se was 1.877, which confirmed that some Se vacancies existed in the ReSe_2_ sample.

These calculation results of monolayer ReSe_2_ are analogous to that of monolayer ReS_2_ as in ref. 18. Moreover, the bandgap of certain ReSe_2_ configuration is smaller than that of ReS_2_ with the same vacancy concentration which mainly attribute to the distortion of Re–Se bond is larger than that of Re–S bond. It can be concluded that the introduction of Se vacancies in monolayer ReSe_2_ makes it a promising candidate as a broad band saturable absorber.

## 3. Fabrication and optical characteristics of the ReSe_2_-SA

As shown in Fig. 2(b), the ReSe_2_-SA was prepared with LPE method, which has been extensively applied in BP and other 2D materials^17–19,32^ fabrication. This method enables large-scale exfoliation and uniform dispersion in the exfoliation medium. First, the bulk ReSe_2_ with purity of 99.995%, purchased from HQ Graphene Inc, was ground to powder in a mortar. Then, the ReSe_2_ powder was dispersed into ethyl alcohol followed by 4 hours sonication. To avoid feature changes of the ReSe_2_ under high temperature, the ultrasonic process was operated at a suitable interval and ice was added during the ultrasonic process. After that, the as-prepared ReSe_2_ solution was centrifuged at 4000 rpm for 10 min with the aim of removing the large-size ReSe_2_ sheets and the supernatant was collected for standby. Finally, the processed ReSe_2_ solution was spin coated on an output mirror (*T* = 5% @ 1.9–2.1 μm), and dried under an infrared oven lamp.

**Fig. 3:**
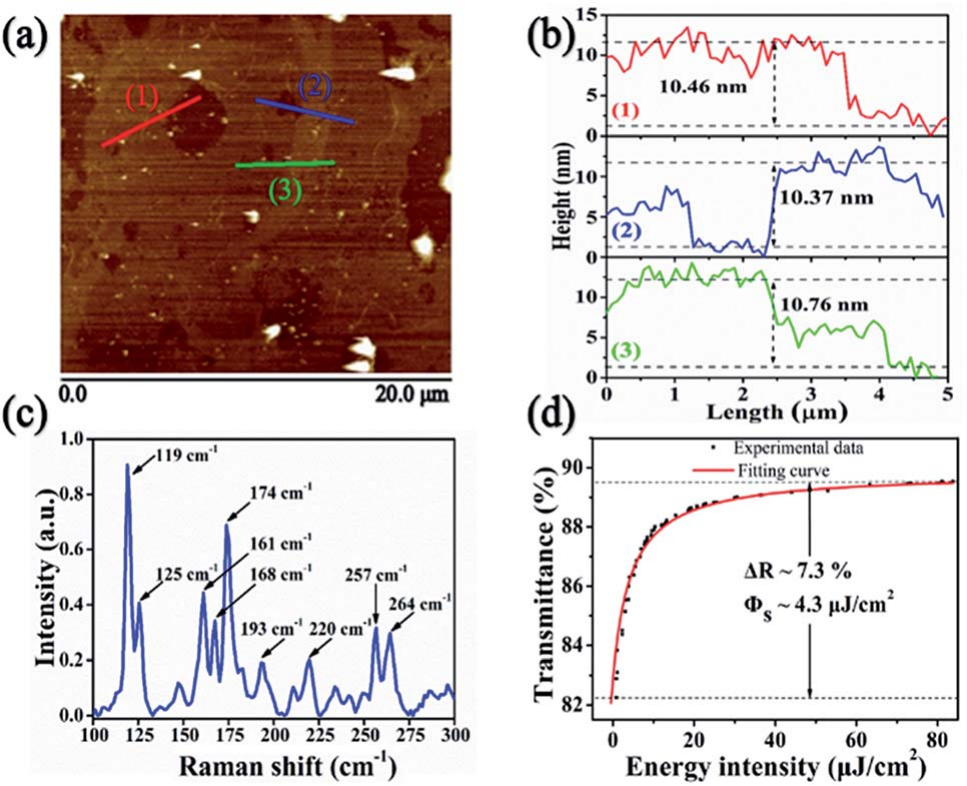
(a) AFM image of ReSe_2_-SA, (b) height variations, (c) Raman spectra of the ReSe_2_-SA, and (d) nonlinear transmission *versus* energy intensity.

In order to investigate the thickness and distribution of the ReSe_2_ flakes, the atomic force microscopy (AFM, Veeco Dimension Icon) was employed to study the morphology of the as-prepared ReSe_2_-SA. Fig. 3(a) shows an AFM scan image of the sample in a large area, because of the weak interlayer coupling of the ReSe_2_. The average thickness of the transferred layers on the treated SAM is approximately 10 nm, as presented in Fig. 3(b), corresponding to the layer number of ∼14 since the height of monolayer ReSe_2_ is 0.7 nm.^24^ The Raman spectra of ReSe_2_-SA was measured by a Raman microscope with a 532 nm laser as the excitation source, as shown in Fig. 3(c). Due to the weak lattice coupling between the layers, these vibrational intense of the low-frequency modes are stronger and the low-frequency modes are easier to be observed. Three typical Raman peaks at 119 cm^−1^, 161 cm^−1^ and 174 cm^−1^, respectively, were observed, which is consistent with the previous reported results.^22,27,29^

The saturable absorption properties of ReSe_2_-SA were also characterized with an open aperture Z-scan method based on a home-made SESAM mode-locked 3 ps Tm:YAP laser at 1940 nm with a repetition rate of 92.5 MHz. The transmittances of the ReSe_2_-SA at different incident fluences were measured and fitted by the following formula:

1 *T*(*F*) = *A*_exp_(−Δ*T*/(1 + *F*/*F*_sat_) + *α*_ns_)

where *T*(*F*) is the transmission rate, *A* is the normalization constant, Δ*T* is the modulation depth, *F* is the input fluence, *F*_sat_ is the saturation fluence and *α*_ns_ is the non-saturable loss. By fitting the curve as shown in Fig. 3(d), the modulation depth and saturation fluence at 2 μm wavelength is 7.3% and 4.3 μJ cm^−2^, respectively.

## 4. Experimental

**Fig. 4:**
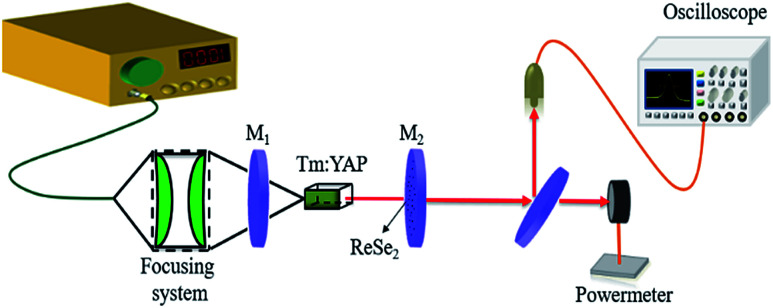
Experimental configuration of PQS Tm:YAP laser.

To investigate the saturable absorption properties of ReSe_2_ in 2 μm region, a compact concave-plano cavity with a length of 24 mm was employed, and the experimental setup is shown in Fig. 4. An high quality a-cut Tm:YAP crystal with both sides high-transmission (HT) coated at 793 nm and 1980 nm was employed as the gain medium. The pump source was a 793 nm fiber coupled LD with a core diameter of 200 μm and a numerical aperture (N.A.) of 0.22. The pump beam was collimated into the Tm:YAP crystal with a spot radius of 100 μm through a 1 : 1 focusing system. The mirror M_1_ was a concave mirror with a curvature radius of 300 mm coated for anti-reflection (AR) at 780–810 nm on the entrance surface, and high-reflectivity (HR) at 1.9–2.1 μm on the cavity surface. The output coupler M_2_ was a flat mirror with a transmission of 5% at around 2 μm. On behalf of removing the heat, the Tm:YAP crystal was wrapped with an indium foil and mounted in a copper block sustained at 17 °C by circulating water. In the measurement of laser output power, a dichroic beamsplitter was applied to dissipate the leaking pump light from the laser cavity.

**Fig. 5:**
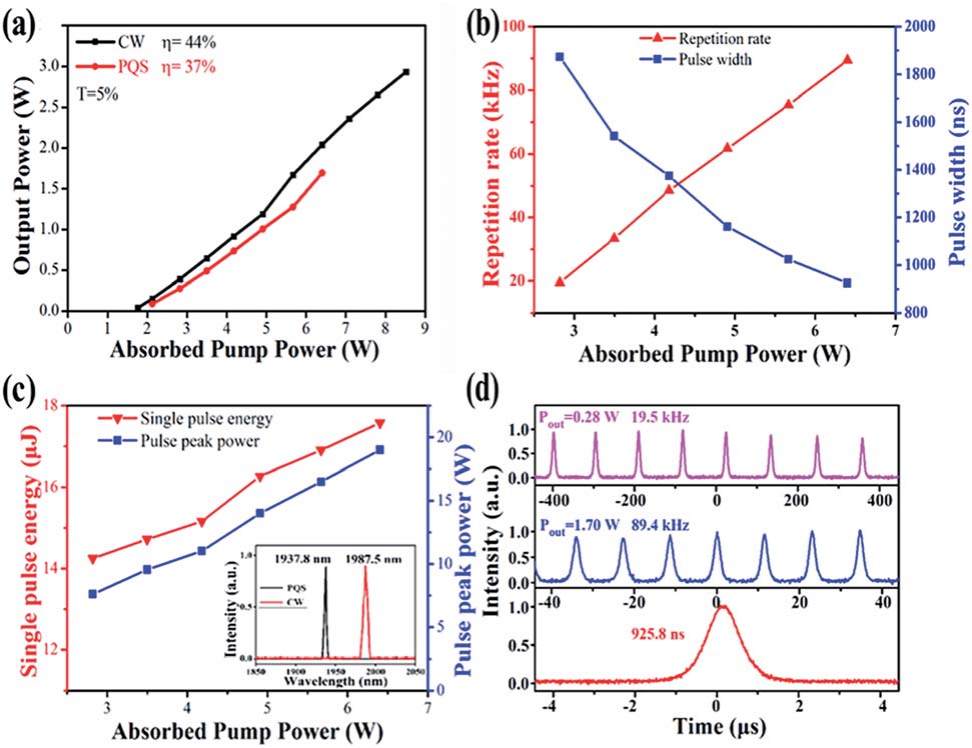
Laser properties *versus* absorbed pump power: (a) CW and PQS output power (b) pulse repetition rate and pulse duration, (c) single pulse energy and peak power; inset: laser spectra, and (d) pulse train and single pulse profile.

First, CW laser operation regime was achieved by using an output mirror (OM, *T* = 5%) without the ReSe_2_-SA. To protect the gain medium, the maximum absorbed pump power was limited to 8.5 W. A maximum CW output power of 2.9 W was obtained with a slope efficiency of 44%. Then, by substituting ReSe_2_ SAM for the plane OM and carefully aligning its position, the PQS operation of the Tm:YAP laser at 2 μm was realized. At the absorbed pump power of 6.4 W, the highest PQS average output power of 1.7 W was achieved, corresponding to a slope efficiency of 37%. While increasing pump power, the ReSe_2_ sample got damaged. Fig. 5(a) shows the output power of the CW and PQS laser operations *versus* the absorbed pump power, respectively. Compared to the CW laser operation, the PQS laser threshold was slightly raised from 1.68 W to 2.55 W and the slope efficiency was dropped from 44% to 37% on account of insertion loss.

**Fig. 6:**
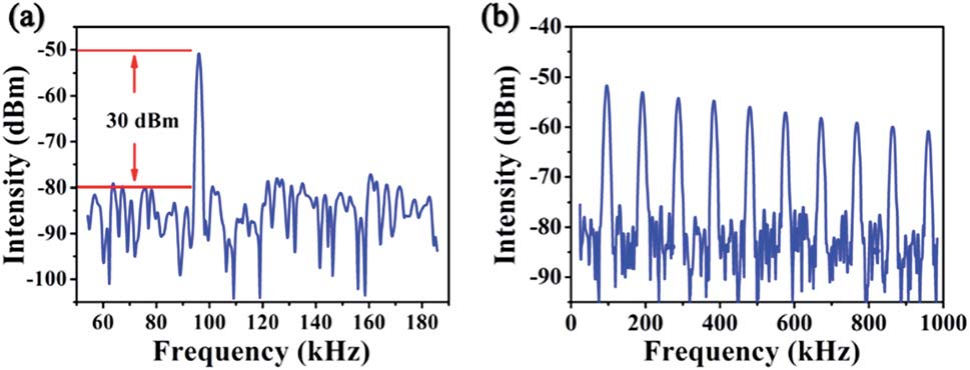
The radio frequency spectra of the Q-switched pulses.

The Q-switched pulse trains and profiles were detected by a fast InGaAs photodetector (Newport, Model 818-BB-51) with a rise time of 35 ps and recorded with a digital oscilloscope (Tektronix DPO 7104C, 1 GHz bandwidth, 20 Gs s^−1^ sampling rates). Fig. 5(b) presents the pulse repetition rate and width as a function of absorbed pump power. In the stable PQS regime, the pulse repetition rate and width varied from 19.5 kHz to 89.4 kHz and 1.8 μs to 925.8 ns, respectively. Under the highest absorbed pump power, the maximum pulse energy was reached to 17.6 μJ, corresponding to the maximum peak power of 19 W. The relationships between the pulse energy and peak power on the absorbed pump power are described in Fig. 5(c). The measured spectrum was inset in Fig. 5(c), it obviously illustrates that the central wavelength of CW laser is 1987.5 nm while it is 1937.8 nm for the Q-switched laser. The blueshift phenomenon were might be due to the high intracavity loss introduced by the ReSe_2_-SA. The typical pulse width of 925.8 ns and pulse repetition rate of 89.4 kHz are shown in Fig. 5(d). Fig. 6 shows the radio frequency (RF) spectrum of the output laser, and the signal-to-noise ratio (SNR) of the first beat was 30 dB at 89.4 kHz, indicating that the Q-switched laser pulse was relatively stable.

Table 1 summarizes the 2 μm PQS Tm:YAP laser results ever achieved by employing nanomaterials as SAs. The BP Q-switched 2 μm laser in ref. 33 had higher peak power, pulse energy and shorter pulse width. However, the shortcoming of instability in air limited the application of BP in generating long-time stable pulsed laser. Compared with the other Nano-SAs, such as graphene, MoTe_2_, MoS_2_ and ReS_2_, our results could be considered superior than that. We believe that a better laser performance would be achieved by further optimizing the parameters of laser cavity and the characteristics of ReSe_2_-SA.

**Table 1:** Comparison of the Tm:YAP (or Tm,Ho:YAP) 2 μm laser output characteristics with Nano-SAs

Materials	SA	Pout (mW)	Slope efficiency	Pulse width (ns)	PRR (kHz)	Peak power (W)	Pulse energy (μJ)
Tm:YAP^9^	Graphene	362	6.8%	735	42.4	11.6	8.5
Tm,Ho:YAP^17^	MoS_2_	270	5%	435	55	11.3	4.9
Tm:YAP^19^	MoTe_2_	1210	34%	380	144	22.2	8.4
Tm:YAP^33^	BP	3100	48%	181	81	218	39.5
Tm:YAP^34^	ReS_2_	245	19%	415	67.7	8.72	0.36
**Tm:YAP (this work)**	**ReSe**_**2**_	**1700**	**37%**	**925**	**89.4**	**19.0**	**17.6**

## 5. Conclusions

In summary, the band structure of the ReSe_2_ with different ratios of Re and Se was calculated by VASP and the results were that Se atomic defects have the tendency to reduce the bandgap. The few-layered ReSe_2_-SA was prepared by LPE method and the saturable absorption effect was characterized around 2 μm. Using the as-prepared ReSe_2_-SA, a PQS 2 μm Tm:YAP laser was demonstrated for the first time, to the best of our knowledge. A maximum PQS average output power of 1.7 W was obtained, corresponding to an optical-to-optical and a slope conversion efficiency of 26.6% and 37%, respectively. The shortest pulse duration of 925.8 ns was realized with a pulse repetition rate of 89.4 kHz, resulting in the maximum single pulse energy and peak power of 17.6 μJ and 19.0 W, respectively. The results indicate that the feasibility of layered ReSe_2_ as SA for achieving high-efficient 2 μm pulsed lasers.

## Conflicts of interest

There are no conflicts to declare.

## Supplementary Material
